# Genome Sequence of *Lactobacillus plantarum* Strain UCMA 3037

**DOI:** 10.1128/genomeA.00251-13

**Published:** 2013-05-23

**Authors:** Saima Naz, Raouf Tareb, Marion Bernardeau, Melissa Vaisse, Celine Lucchetti-Miganeh, Mathias Rechenmann, Jean-Paul Vernoux

**Affiliations:** Research Unit EA 4651, Aliments Bioprocédés Toxicologie Environnements (ABTE), Université de Caen Basse-Normandie, Esplanade de la paix, Caen, Francea; Genostar, Montbonnot, Franceb

## Abstract

Nucleic acid of the strain *Lactobacillus plantarum* UCMA 3037, isolated from raw milk camembert cheese in our laboratory, was sequenced. We present its draft genome sequence with the aim of studying its functional properties and relationship to the cheese ecosystem.

## GENOME ANNOUNCEMENT

*Lactobacillus plantarum* is a lactic acid bacterium commonly found in numerous ecological niches such as vegetables, meat, fish, and dairy products (1). In dairy products, it is usually present in milk and cheeses as an adjunct starter or as a nonstarter lactic acid bacterium (NSLAB). *Lactobacillus plantarum* strain UCMA 3037, isolated in our laboratory from raw milk camembert cheese, is a probiotic candidate strain and is capable of surviving in various cheese matrices (2). For exploring its potentially functional and probiotic properties in detail, we sequenced its genome using high-throughput Illumina/paired-end technology.

The uncompleted draft genome sequence of *L. plantarum* UCMA 3037 was determined by paired-end sequencing using the Illumina GAIIx platform (6,570,144 reads) (Baseclear, Netherlands) with a coverage of >100×.The paired-end reads were assembled *de novo* using the CLC Genomics workbench 5.0 (CLC Bio). The resulting assembly consists of 92 contigs. The draft genome, of about 3.11 Mb, has a G+C content of 44.5%. These data are comparable to those of already reported sequenced genomes of *L. plantarum* strains (1).

At the time of genome submission to GenBank, genome annotation was requested to be performed by NCBI’s Prokaryotic Genomes Annotation Pipeline (PGAAP), which predicted 2,932 protein coding sequences (CDSs).

An internal annotation of the assembled contig sequences was performed with the MetabolicPathwayBuilder *s*ystem (Genostar). MetabolicPathwayBuilder, together with the reference MicroB database, is integrated bioinformatics interactive software dedicated to microbial genome analysis and comparison. MicroB integrates and updates data from several databases, i.e., NCBI Reference Sequence, UniProtKB, ENZYME, Gene Ontology, and KEGG. The GenoAnnot module was used to annotate our *L. plantarum* UCMA 3037 genome. A variant of the PRIAM algorithm (3) was used to predict the enzymatic activities of the proteins.

Within the reconstructed metabolic network, several pathways are representative of carbohydrate metabolism, protein and amino acid metabolism, nucleic acid metabolism, and fermentation metabolism, which correspond to 115 KEGG metabolic pathways. The products of some genes are found to confer an advantage for adaptation to a cheese matrix; for example, malate lactate dehydrogenase (4) and histidine kinase for resistance to an acidic environment (5), two types of glycine/betaine transporter for osmoregulation (6), lipase/esterases for the production of cheese flavor compounds (7), and the serine protease HtrA domain (putative caseinase) (8).

*L. plantarum* UCMA 3037 has been predicted to carry two plasmids: plasmid NC11101, which shares a 64% sequence similarity to the *L. plantarum* plasmid pLTK13 (9), and plasmid NC06278, which shares a 96% sequence similarity to *L. plantarum* plasmid p256 (10). *L. plantarum* UCMA 3037 carries prophage elements similar in sequence to Lp1, Lp2, Lp3, and Lp4 prophages present in reference strains *L. plantarum* WCFS1 and JDM1 (11, 12).

A more detailed analysis is in progress in which we are evaluating the stability, safety, and functional and metabolic aspects of this strain and its relationship to the cheese ecosystem.

### Nucleotide sequence accession numbers.

This whole-genome-shotgun project has been deposited at DDBJ/EMBL/GenBank under the accession number APHP00000000. The version described in this paper is the first version, APHP00000000.1.
